# Adipose Tissue in Multiple Symmetric Lipomatosis Shows Features of Brown/Beige Fat

**DOI:** 10.1007/s00266-020-01666-6

**Published:** 2020-03-10

**Authors:** Daniel Schiltz, Sebastian Tschernitz, Christine Ortner, Alexandra Anker, Silvan Klein, Oliver Felthaus, Niklas Biermann, Julia Schreml, Lukas Prantl, Stephan Schreml

**Affiliations:** 1 Department of Plastic, Hand- and Reconstructive Surgery, University Medical Center, Regensburg, Germany; 2 Department of Dermatology, University Medical Center Regensburg, Franz-Josef-Strauss-Allee 11, 93053 Regensburg, Germany; 3 Institute of Human Genetics, University Hospital of Cologne, Cologne, Germany

**Keywords:** Multiple symmetric lipomatosis (MSL), Morbus Madelung, Launois–Bensaude syndrome, Adipocytes, Brown fat, Beige fat

## Abstract

**Introduction:**

Multiple symmetric lipomatosis (MSL) (syn.: Launois–Bensaude Syndrome, benign symmetric lipomatosis) is a rare disease of fatty tissue. The pathophysiology of MSL still remains unclear, although several approaches have been described in order to understand it. Beside morphological characteristics and some molecular cell biological approaches, little is known about the histological and immunohistochemical characterization of adipose tissue from patients with MSL.

**Methods:**

From the 45 patients with MSL in our database, 10 were included in the study. Fat tissue samples were collected from affected and unaffected areas. The forearm served as a control area as this area is not affected in MSL. The specimens were analyzed after selected stainings were taken (hematoxylin–eosin = HE, Elastica van Gieson, Ladewig, CD200, CIDEA, myf5, p107, Prdm16, Sca-1, syndecan, UCP1, MAC387, Glut4).

**Results:**

In patients suffering from MSL, no macroscopic or microscopic morphological difference could be found between affected and unaffected adipose tissue in HE stainings. The majority of samples showed positivity for UCP1 (9/10 clinically affected tissues, 7/10 clinically unaffected tissues) and CD200.

**Conclusion:**

Marker profiles support the hypothesis that affected adipose tissue derives from brown or beige adipose tissue rather than from white fat.

**Level of Evidence IV:**

This journal requires that authors assign a level of evidence to each article. For a full description of these Evidence-Based Medicine ratings, please refer to the Table of Contents or the online Instructions to Authors www.springer.com/00266.

**Electronic supplementary material:**

The online version of this article (10.1007/s00266-020-01666-6) contains supplementary material, which is available to authorized users.

## Introduction

Multiple symmetric lipomatosis (MSL) (syn.: Launois–Bensaude syndrome, benign symmetric lipomatosis) is a rare disease of fatty tissue. It is defined as a benign adipose tissue hyperplasia characterized by large and symmetric accumulations of fatty tissue [1]. These fatty tissue hyperplasia are unaffected by diet and are even resistant to tumor cachexia [2]. Prevalence of MSL is indicated as 1 in 25,000 [3, 4]. The male-to-female ratio is unclear and is estimated to be between 15 and 30:1 to 1:2 for male:female [3–7].

**Fig. 1:**
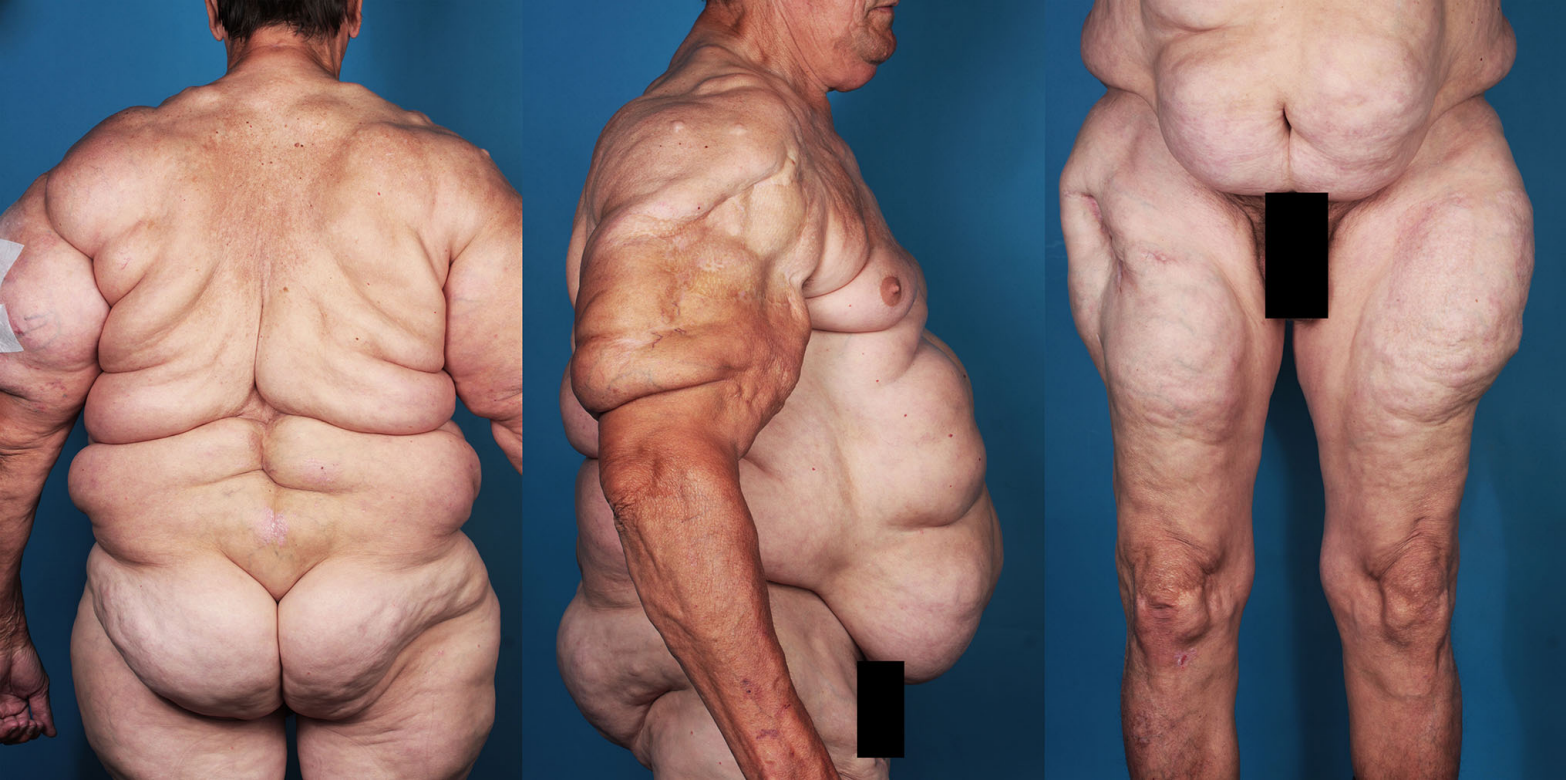
Patient suffering from MSL type III

Diagnosis is established through physical examination and anamnesis. To describe the phenotype, several classifications are used in the literature. The most common classification was established in 1991 by Donhauser and divides patients into four types of MSL [8]. A revised classification was published recently by our research team and divides patients into five types [4] (Table S1, Fig. 1).

The pathophysiology of MSL still remains unclear, although several approaches have been described to understand it. The disease occurs predominantly sporadically, but there have been increasing numbers of familial accumulations that show a dominant inheritance pattern and indicate a monogenic cause [9]. Laboratory and functional studies indicate a change in the response of catecholamine receptors and an increased uptake of fatty acids into the tissue as well as changes in the physiological processes of cellular energy production. For example, myoclonic epilepsy with ragged red fibers, a disorder of the mitochondrial energy metabolism, is frequently associated with lipomatosis [10]. A correlated gene mutation has not been discovered. An impairment of processes in the field of mitochondrial energy production and the fatty acid oxidation in peroxisomes is discussed in [4, 11].

Besides morphological characteristics and some molecular cell biological approaches, little is known about the histological characterization of adipocytes from MSL-affected patients. Specifically, a systematic histological and immunohistological workup and comparison of affected and unaffected adipose tissue in patients with MSL have not previously been performed. In this article, we present data on this specific topic. Based on previous publications, we selected particularly suitable markers and did a histological and immunohistological characterization of affected and non-affected adipose tissue from patients with MSL.

## Materials and Methods

### Patients

We found 45 patients diagnosed with MSL between 2011 and 2017 in the database of the University Hospital, Regensburg. Diagnosis of MSL was clinically conducted by considering morphological characteristics and patient history. Morphological characteristics were defined as disproportionate hypertrophic symmetric fat tissue growth of MSL-typical areas. Anamnestic factors were the sudden occurrence of fat deposits that grew independently of general body fat increase and resistance to conservative therapies, such as diets.

### Tissue Samples

Fatty tissue samples were collected from affected and unaffected areas of the patients (with patient approval). The forearm was chosen as the unaffected area, as this area is never affected by adipose tissue hyperplasia in MSL patients [4]. For patients who did not want to have a scar on the visible forearm, samples were taken from the groin. All patients showed adipose tissue hyperplasia in the shoulder and upper arms region. Therefore, the cranial dorsolateral part of the upper arm was chosen as the affected area. Samples were collected via liposuction or excisions of approximately 1 cm^3^ of subcutaneous fat tissue. Both procedures were performed under local anesthesia (Xylonest^®^1%, Braun, Germany) at the Department of Plastic, Hand- and Reconstructive Surgery, University Hospital, Regensburg, Germany. Fat tissue samples were immediately fixed in 4% formaldehyde solution and later worked up in paraffin.

### Histology

From each paraffin block, 4 μm thick sections were cut off by microtome. For electron microscopic examinations, the material was fixed in a Karnofsky glutaraldehyde solution. For morphological examinations of the adipocytes, hematoxylin–eosin staining was used. In order to visualize the connective tissue histologically, Elastic van Gieson staining and Ladewig staining were used. For the discrimination of brown, white and beige adipose tissue, the markers CD200, CIDEA, myf5, p107, Prdm16, Sca-1, syndecan and UCP1 were used immunohistochemically. To classify the inflammatory infiltrate, the immunohistochemical marker MAC387 was used. Glut4 was used to visualize the glucose transport into the cell. Table S2 summarizes the meaning of these stains. The detailed protocols of all stainings are listed in the supplement.

### Ethics

The study was approved by the Ethical Committee of the University of Regensburg and Cologne (reference number Regensburg: 08/117 and 14-101-138. Reference number Cologne: 13-142).

## Results

### Patient Characteristics

Of the 45 patients suffering from MSL, samples were collected from 10 Patients (*n* = 10). From 7 of these, fatty tissues of affected and unaffected areas could be obtained. From the remaining 3 patients, only fatty tissue from affected area was obtained (patients’ choice). Four patients showed the phenotype Ib, 5 patients were classified as a type Ic, and one patient showed the phenotype III according to the latest classification [4]. Five patients were female, and 5 were male. The mean age of the patients at time of inclusion was 59 years (range 39–77 years).

### Histology

**Fig. 2:**
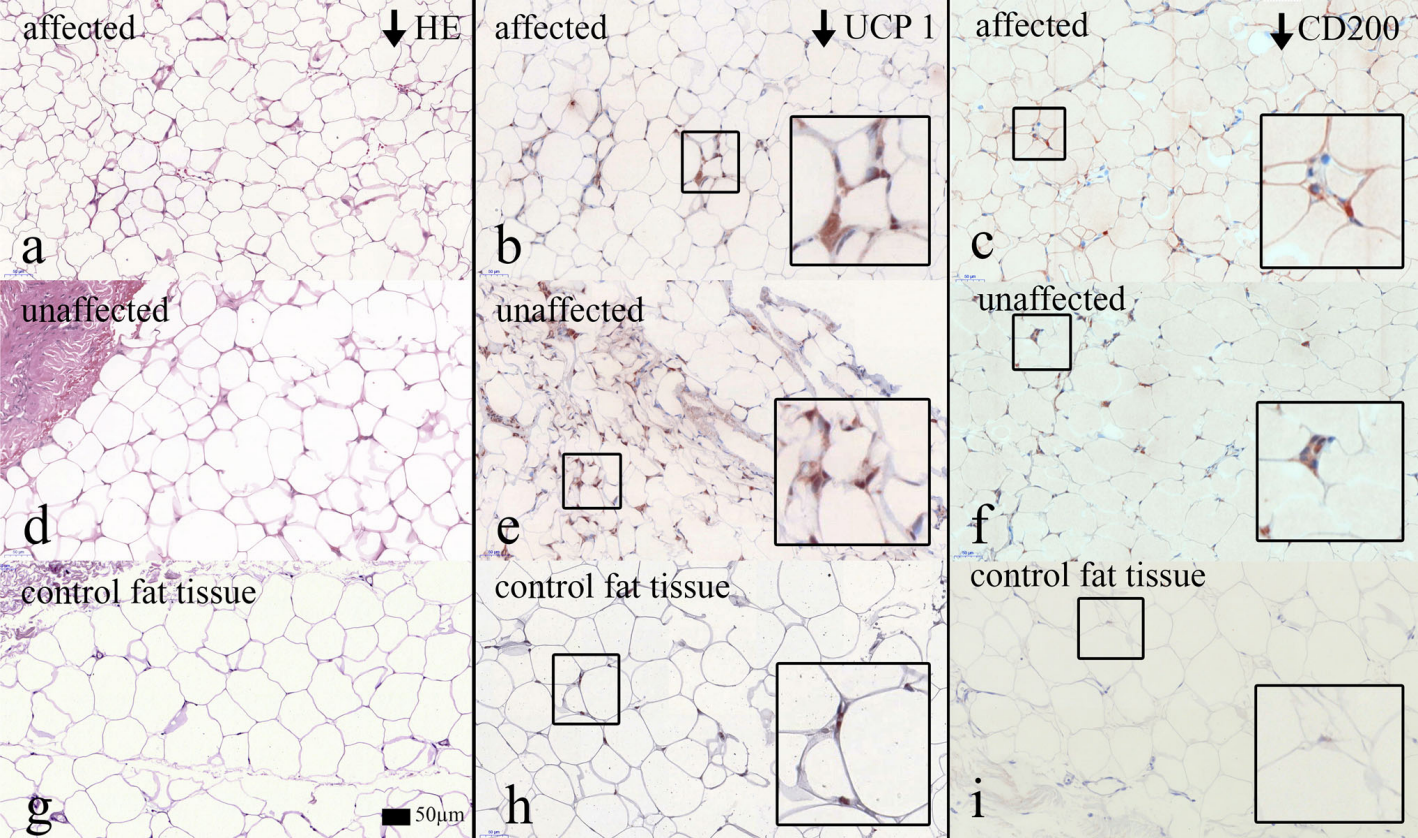
Histology and Immunohistochemistry. **a** Hematoxylin–eosin staining of affected fat tissue; **b** UCP1 staining of affected fat tissue; **c** CD200 staining of affected fat tissue; **d** hematoxylin–eosin staining of unaffected fat tissue; **e** UCP1 staining of affected fat tissue; **f** CD200 staining of unaffected fat tissue; **g** hematoxylin–eosin staining of control fat tissue; **h** UCP1 staining of control fat tissue; **i** CD200 staining of control fat tissue. Sample images from patient nr. 10

In the hematoxylin–eosin staining (HE staining), univacuolar (white) adipose tissue was seen in both affected and unaffected adipose tissue. There was no evidence of beige or brown adipose tissue histologically. Elastic van Gieson and Ladewig staining showed no increase in collagen or elastic fibers (Figure 2).

### Immunohistochemistry

All affected tissue (*n* = 10) and unaffected tissue (*n* = 7) were immunohistochemically examined. In the affected adipose tissue, 9 patients tested positive for UCP1. In one assay only, a partial positive test for UCP1 was found. In the unaffected adipose tissue, all examined patients tested positive for UCP1 (Fig. 2). CD 200 was equivalent to UCP1, that is, being partially weakly positive in the affected adipose tissue as well as in the unaffected adipose tissue (Fig. 2). In 4 assays, a partially very weak positive test for myf5 was found in the affected adipose tissue. The other 6 assays of affected adipose tissue were negative for myf5. In a single assay, a partially very weak positive test for myf5 was found in the unaffected adipose tissue. The remaining stains were negative for this marker. Sca1 was partially weakly positive for the affected adipose tissue and consistently negative in unaffected adipose tissue. Neither in the affected nor in the unaffected fatty tissue could CIDEA, p107, Prdm16 or syndecan be detected by immunohistochemistry. Only glut 4 was once partially positive in unaffected adipose tissue. In none of the cases was it possible to detect an increased number of macrophages using the marker MAC387. Table 1 summarizes the results. An example of all stainings from patient no. 10 is shown in Figure S1 in the supplementary material.

**Table 1:**
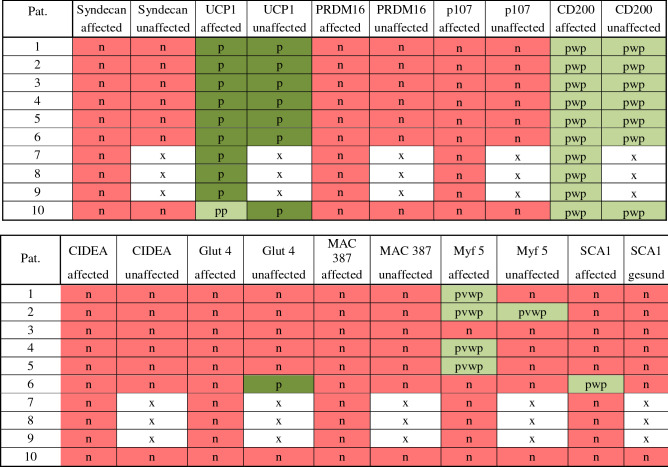
Results of immunohistochemically cell examination: n = negative; p = positive; pp = partially positive; pwp = partially week positive; pvwp = partially very week positive; x = missing data

## Discussion

There are only a few microscopic descriptions of affected adipose tissue in MSL patients in the literature. A systematic histological and immunohistological workup of affected and unaffected adipose tissue in more than one MSL patient has never been previously been performed.

The hematoxylin–eosin staining did not show any differences between the affected and unaffected fatty tissue. This agrees with the findings of some case reports, where macroscopically regular lobulated adipose tissue without any abnormalities was described in samples of affected fatty tissue from MSL patients [12–14]. Although Agostini et al. [15] compared affected adipose tissue with unaffected adipose tissue in one patient with MSL and described dystrophic adipocytes with significantly enlarged fat vacuoles (30%) in the affected tissue, Pollock et al. [16] described a diffuse spread of adipose tissue along nerve vein sheaths as well as in muscle compartments.

The immunohistochemical cell examinations showed the most promising results. Beige fatty tissue is recruited from the same progenitor cells as white fat and, similar to brown adipose tissue, expresses UCP1 (= uncoupling protein 1). Physiologically, UCP1 is expressed in the inner mitochondrial membrane of brown adipose tissue and serves to generate heat. The stimulus takes place by a release of norepinephrine from nerve fiber endings, which, binding to the β3 adrenoreceptor, leads to the decoupling of oxidative phosphorylation [17]. The constitutive brown adipose tissue probably originates from a myogenic progenitor cell line and expresses the transcription factor myf5. The formation of inducible beige adipose tissue within the subcutaneous white adipose cell deposit is subject to on-demand control (e.g., cold irritation) [18]. Nisoli et al. [19] also reported over-expression of UCP1 in the affected adipose tissue in MSL patients, but no over-expression of UCP1 in the unaffected adipose tissue of the same patients. These studies were performed on cell cultures. In contrast, we were able to detect immunohistochemically the expression of UCP1 also in unaffected adipose tissue (negative reference fat tissue) in our group. This suggests that not only the affected adipose tissue of MSL patients, but also the unaffected adipose tissue carries genetic alterations and would explain the progression of MSL in previously unaffected areas of the body. In contrast, Kazumi et al. [12] were not able to detect any expression of UCP1 in the affected adipose tissue in an MSL patient. In a previous publication, we were already able to show an increase in UCP1 in real-time reverse-transcriptase polymerase chain reaction analysis [12].

CD200 (Cluster of Differentiation 200) or OX-2 membrane glycoprotein is a type I membrane glycoprotein. It has two extracellular immunoglobulin domains, a transmembrane and a cytoplasmic domain. It belongs to the immunoglobulin superfamily. CD200 is expressed in different cell types and plays an important role in immunosuppression and regulation of anti-tumor activity. Studies indicate that this gene can regulate myeloid cell activity and provide an inhibitory signal for the macrophage lineage in various tissues. (UniProt. Retrieved 16 May 2013). The staining was weakly positive in subcutaneous adipose tissue of the MSL patients. In control fatty tissue, the staining was negative (Fig. 2). CD200 is expressed by poorly differentiated white adipose tissue [20]. This might also indicate that adipose cells from MSL patients are in an early stage of differentiation, such as brown or beige fat.

Prdm16 controls the differentiation to muscle or brown adipose tissue. Loss of Prdm16 from brown-cell precursor cells promotes further differentiation into muscle cells [21]. An over-expression of Prdm16 in the affected adipose tissue could not be confirmed in our investigations. The reason for this may be the limited sensitivity in immunohistochemical staining.

Myf5 belongs to the family of “myogenic regulatory factors” (MRFs) and is a “bHLH (basic helix loop helix) transcription factor” [22]. Myf5 (myogenic factor 5) is a protein that plays a key role in the regulation of muscular differentiation (myogenesis of skeletal muscle) and development of brown adipose tissue. This transcription factor is only expressed in embryonic tissue for a few days. White and brown fat cells emerge from different progenitor cells during embryonic development. Brown fat arises from cells that express myf5 and can also differentiate into skeletal muscle. White fat develops from mesodermal myf5-negative stem cells [23]. Stainings of all the tested patients in our study were negative to myf5.

Scime et al. [24] show that mice lacking p107 showed a uniform replacement of white adipose tissue with brown adipose tissue. p107 (retinoblastoma-like protein 1, RBL1) is a tumor suppressor protein that appears to be involved in cell cycle regulation. It is similar to the product of the retinoblastoma 1 gene (RB1) and is phosphorylated in the S and M phase of the cell cycle and dephosphorylated in the G1 phase of the cell cycle [25]. It has been demonstrated that it plays a role in adipocyte differentiation and is necessary for the differentiation of white adipose tissue [24, 26]. Although all samples tested in this study were negative to p107.

The cell death activator CIDEA is an essential transcriptional coactivator that regulates the milk gland secretion of milk lipids. It has also been shown to activate apoptosis. Mice lacking functional CIDEA has higher metabolic rates, higher lipolysis in brown adipose tissue and higher body core temperatures when exposed to cold. These mice are also resistant to diet-induced obesity and diabetes [27, 28]. None of our samples was positive for CIDEA.

Syndecan is a lipoprotein uptake receptor and is expressed in a variety of different tissue types [29]. In knockout mice without syndecan, the intraepidermal fat layer is only weakly formed [30]. An immunological examination of the affected tissue does not exist. No reference in the literature or in our investigation could be found regarding this.

GLUT-4 (type 4 glucose transporter) is a protein that is particularly localized in vesicles in mammalian cells. GLUT-4 is a membrane transport protein. In humans, GLUT-4 is expressed in striped muscle and fat cells (UniProt P14672). We did not see any indications of an increased glucose turnover. An upregulation of the GLUT-4 receptor in the adipose tissue was not seen.

## Conclusion

In patients suffering from MSL, no microscopic differences could be found between affected and unaffected adipose tissue. The proliferation of connective tissue and vessels as an indicator of tissue activity could not be found. We therefore consider the proliferation of adipose tissue as tissue hyperplasia and not as tissue hypertrophy. UCP1 was significantly more highly expressed in almost all MSL patients (affected and unaffected adipose tissue) than in healthy patients. The immunohistochemical marker profiles (UPC1 and CD200) indicate that affected adipose tissue could derive from brown or beige adipose tissue rather than from white fat.

## Electronic Supplementary Material

Below is the link to the electronic supplementary material.

**Figure S1**: overview of all stainings of patient nr. 10 (JPEG 19226 kb)

Supplementary material 2 (DOCX 13 kb)

Supplementary material 3 (DOCX 13 kb)

Supplementary material 4 (DOCX 29 kb)
