# Supplementary figures and images for: Adipose Tissue in Multiple Symmetric Lipomatosis Shows Features of Brown/Beige Fat
Source: Aesthetic Plast Surg. 2020 Mar 10;44(3):855–61. doi: 10.1007/s00266-020-01666-6 (PMC7280331; doi:10.1007/s00266-020-01666-6)

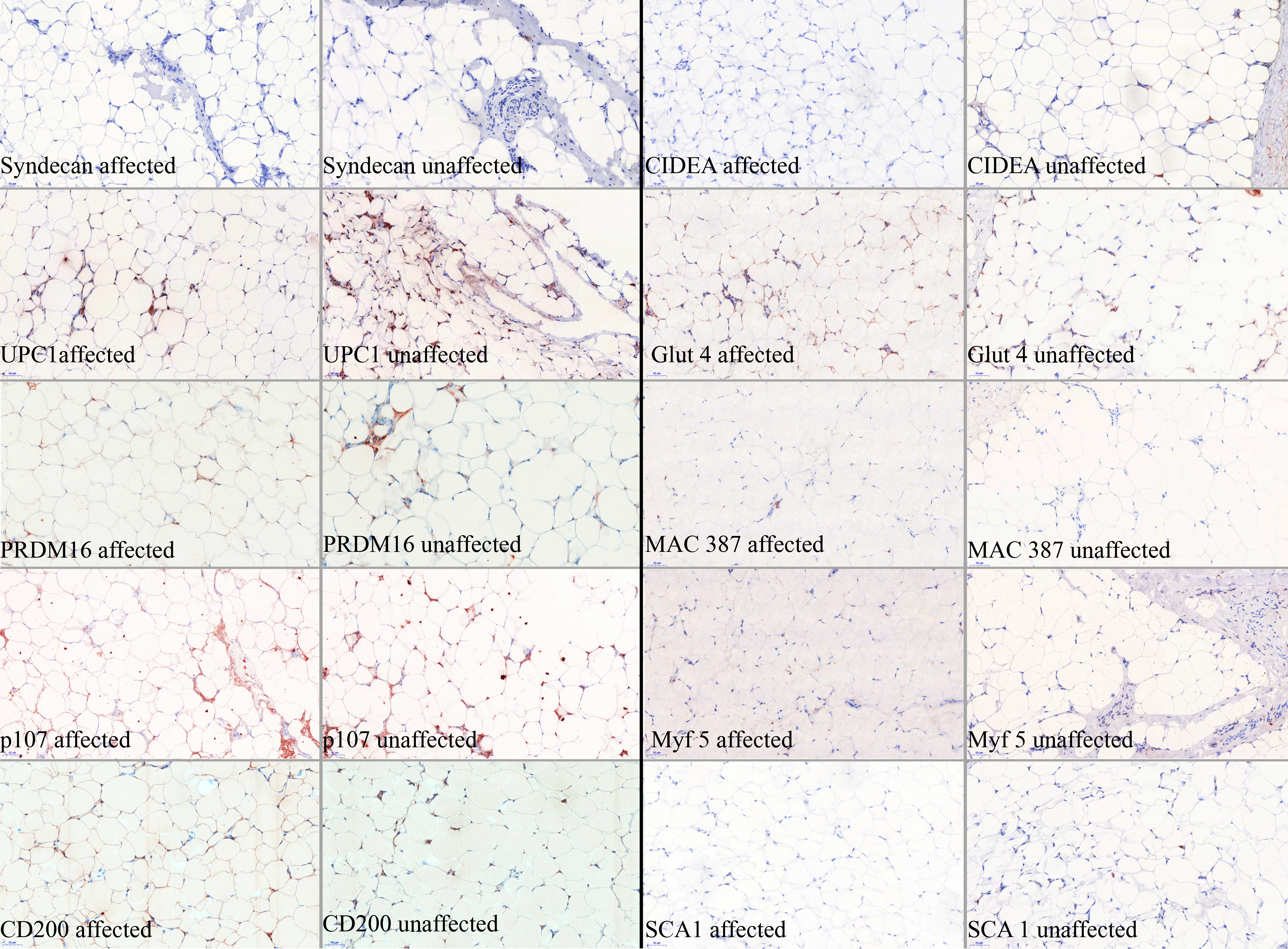

Supplement: Supplementary file 1 — Figure S1: overview of all stainings of patient nr. 10 (JPEG 19226 kb) [file 266_2020_1666_MOESM1_ESM.jpg]
