# Supplementary material for: Adipose Tissue in Multiple Symmetric Lipomatosis Shows Features of Brown/Beige Fat
Source: Aesthetic Plast Surg. 2020 Mar 10;44(3):855–61. doi: 10.1007/s00266-020-01666-6 (PMC7280331; doi:10.1007/s00266-020-01666-6)
Supplement: Supplementary file 2 — Supplementary material 2 (DOCX 13 kb) [file 266_2020_1666_MOESM2_ESM.docx]

| **Phenotypes** | | **Affected body areas** |
| --- | --- | --- |
| Type I | Ia | Neck |
|  | Ib | Neck, shoulder girdle, upper arms |
|  | Ic | Neck, shoulder girdle, upper arms, chest, abdomen, upper and lower back |
| Type II | | Hips, bottom and upper legs |
| Type III | | General distribution skipping head, forearms and lower legs |

**Table S1:** Phenotypes of MSL according to Schiltz et al. [4]
