# Supplementary material for: Adipose Tissue in Multiple Symmetric Lipomatosis Shows Features of Brown/Beige Fat
Source: Aesthetic Plast Surg. 2020 Mar 10;44(3):855–61. doi: 10.1007/s00266-020-01666-6 (PMC7280331; doi:10.1007/s00266-020-01666-6)
Supplement: Supplementary file 3 — Supplementary material 3 (DOCX 13 kb) [file 266_2020_1666_MOESM3_ESM.docx]

| **staining** | **significance** |
| --- | --- |
| Hämatoxylin-Eosin | morphology |
| Elastik van Geison | connective tissue |
| Ladewig | connective tissue |
| CD 200 | expression of poorly differentiated white adipose tissue |
| CIDEA | expression of white adipose tissue |
| myf5 | expression of brown adipose tissue |
| p107 | expression of precursor cells of white adipose tissue |
| Prdm16 | expression of beige/ brown adipose tissue |
| Sca-1 | expression of precursor cells of brown adipose tissue |
| Syndecan | expression of precursor cells of adipose tissue |
| UCP1 | expression of beige/ brown adipose tissue  thermogenic marker |
| MAC387 | expression of monocytes and macrophages |
| Glut4 | Glucose transporter |

**Table S2:** Overview of the used stainings
