# Supplementary material for: Adipose Tissue in Multiple Symmetric Lipomatosis Shows Features of Brown/Beige Fat
Source: Aesthetic Plast Surg. 2020 Mar 10;44(3):855–61. doi: 10.1007/s00266-020-01666-6 (PMC7280331; doi:10.1007/s00266-020-01666-6)
Supplement: Supplementary file 4 — Supplementary material 4 (DOCX 29 kb) [file 266_2020_1666_MOESM4_ESM.docx]

**Supplements**

**Immunohistochemical protocols:**

**Syndecan 1**

**Reagents:**

- Anti- Syndecan 1 antibody (Abcam)
- Citrate Buffer, (Zytomed)
- Dulbecco´s Phosphate Buffered Saline (Sigma-Aldrich)
- Hydrogen peroxide 100 volumes >30%, (Fisher Scientific)
- ZytoChemPlusHRP Kit/Rabbit (Zytomed)
- Antibody Diluent (Zytomed)
- Auqatex (Merck)
- Hämalaun sauer nach Mayer (Roth)
- Xylol (Merck)
- Ethanol

**Implementation:**

- positive control/localization: tonsils, lymph nodes, coloncarcinoma / membrane staining
- let paraffin drain in the heat cabinet at 72°C for 30min
- 2 x Xylol for 10 min.; 2 x 100%/ 96%/ 70% ethanol for 5 min.; wash in distillated aqua
- samples for ½ h in Citrate Buffer pH6; cook in a steamer for ½ h
- cool down samples for 20 min.
- samples for 10min. in 3% Hydrogen Peroxide / wash in distillated aqua
- wash samples for 6min. in Dulbecco´s Phosphate Buffered Salin
- samples in protein-block (ZytoChemPlusHRP Kit/Rabbit) for 10min
- wash samples for 6min. in Dulbecco´s Phosphate Buffered Salin
- ad primary antibody Syndecan 1, dilution 1: 100, let it over night at 4°C in AntibodyDiluent
- wash samples for 6min. in Dulbecco´s Phosphate Buffered Salin
- 15min. secondary antibody; ZytoChemPlusHRP Kit/Rabbit
- wash samples for 6min. in Dulbecco´s Phosphate Buffered Salin
- 15min. enzym-konjugat; ZytoChemPlusHRP Kit/Rabbit
- wash samples for 6min. in Dulbecco´s Phosphate Buffered Salin
- AEC dye, microscopic control after 5-15min / stop reaction in distillated aqua
- counter-dyeing and blue with “Mayers Hämalaun”
- cover with “Auqatex“

**UCP1**

**Reagents:**

- Anti- UCP1 antibody (Abcam)
- Citrate Buffer, (Zytomed)
- Dulbecco´s Phosphate Buffered Saline (Sigma-Aldrich)
- Hydrogen peroxide 100 volumes >30%, (Fisher Scientific)
- ZytoChemPlusHRP Kit/Rabbit (Zytomed)
- Antibody Diluent (Zytomed)
- Auqatex (Merck)
- Hämalaun sauer nach Mayer (Roth)
- Xylol (Merck)

**Implementation:**

- Positiv Kontrolle/Lokalisation: coloncarcinoma/ cytoplasmatic
- let paraffin drain in the heat cabinet at 72°C for 30min
- 2 x Xylol for 10 min.; 2 x 100%/ 96%/ 70% ethanol for 5 min.; wash in distillated aqua
- citrate Buffer pH6 for ½ h, cook in a steamer for ½ h
- samples for ½ h in Citrate Buffer pH6; cook in a steamer for ½ h
- cool down samples for 20 min.
- samples for 10min. in 3% Hydrogen Peroxide / wash in distillated aqua
- wash samples for 6min. in Dulbecco´s Phosphate Buffered Salin
- samples in protein-block (ZytoChemPlusHRP Kit/Rabbit) for 10min
- wash samples for 6min. in Dulbecco´s Phosphate Buffered Salin
- ad primary antibody UCP 1, dilution 1: 100, let it over night at 4°C in AntibodyDiluent
- wash samples for 6min. in Dulbecco´s Phosphate Buffered Salin
- 15min. secondary antibody; ZytoChemPlusHRP Kit/Rabbit
- wash samples for 6min. in Dulbecco´s Phosphate Buffered Salin
- 15min. enzym-konjugat; ZytoChemPlusHRP Kit/Rabbit
- wash samples for 6min. in Dulbecco´s Phosphate Buffered Salin
- AEC dye, microscopic control after 5-15min / stop reaction in distillated aqua
- counter-dyeing and blue with “Mayers Hämalaun”
- cover with “Auqatex“

**PRDM16**

**Reagents:**

- Anti-PRDM 16 antibody (Abcam)
- Dulbecco´s Phosphate Buffered Saline (Sigma-Aldrich)
- Hydrogen peroxide 100 volumes >30%, (Fisher Scientific)
- ZytoChemPlusHRP Kit/Rabbit (Zytomed)
- Antibody Diluent (Zytomed)
- Auqatex (Merck)
- Hämalaun sauer nach Mayer (Roth)
- Xylol (Merck)
- Ethanol

**Implementation:**

- positive control/localization: human cerebellum / nucleus
- let paraffin drain in the heat cabinet at 72°C for 30min
- 2 x Xylol for 10 min.; 2 x 100%/ 96%/ 70% ethanol for 5 min.; wash in distillated aqua
- samples for 10min. in 3% Hydrogen Peroxide
- wash samples for 6min. in Dulbecco´s Phosphate Buffered Salin
- samples in protein-block (ZytoChemPlusHRP Kit/Rabbit) for 10min
- wash samples for 6min. in Dulbecco´s Phosphate Buffered Salin
- ad primary antibody PRDM16 (c:2,5µg/ml), dilution 1: 400, let it over night at 4°C in AntibodyDiluent
- wash samples for 6min. in Dulbecco´s Phosphate Buffered Salin
- 15min. secondary antibody; ZytoChemPlusHRP Kit/Rabbit
- wash samples for 6min. in Dulbecco´s Phosphate Buffered Salin
- 15min. enzym-konjugat; ZytoChemPlusHRP Kit/Rabbit
- wash samples for 6min. in Dulbecco´s Phosphate Buffered Salin
- AEC dye, microscopic control after 5-15min / stop reaction in distillated aqua
- counter-dyeing and blue with “Mayers Hämalaun”
- cover with “Auqatex“

**p107**

**Reagents:**

- Anti- p107 antibody (Abcam)
- Citrate Buffer, (Zytomed)
- Dulbecco´s Phosphate Buffered Saline (Sigma-Aldrich)
- Hydrogen peroxide 100 volumes >30%, (Fisher Scientific)
- ZytoChemPlusHRP Kit/Rabbit (Zytomed)
- Antibody Diluent (Zytomed)
- Auqatex (Merck)
- Hämalaun sauer nach Mayer (Roth)
- Xylol (Merck)
- Ethanol

**Implementation:**

- positive control/localization: lymph nodes, coloncarcinoma / nucleus
- let paraffin drain in the heat cabinet at 72°C for 30min
- 2 x Xylol for 10 min.; 2 x 100%/ 96%/ 70% ethanol for 5 min.; wash in distillated aqua
- samples for ½ h in Citrate Buffer; cook in a steamer at 50°C for ½ h
- cool down samples for 20 min.
- samples for 10min. in 3% Hydrogen Peroxide / wash in distillated aqua
- wash samples for 6min. in Dulbecco´s Phosphate Buffered Salin
- samples in protein-block (ZytoChemPlusHRP Kit/Rabbit) for 10min
- wash samples for 6min. in Dulbecco´s Phosphate Buffered Salin
- ad primary antibody p107, dilution 1: 10, let it over night at 4°C in AntibodyDiluent
- wash samples for 6min. in Dulbecco´s Phosphate Buffered Salin
- 15min. secondary antibody; ZytoChemPlusHRP Kit/Rabbit
- wash samples for 6min. in Dulbecco´s Phosphate Buffered Salin
- 15min. enzym-konjugat; ZytoChemPlusHRP Kit/Rabbit
- wash samples for 6min. in Dulbecco´s Phosphate Buffered Salin
- AEC dye, microscopic control after 5-15min / stop reaction in distillated aqua
- counter-dyeing and blue with “Mayers Hämalaun”
- cover with “Auqatex“

**CD200**

**Reagents:**

- Anti- UPC1 antibody (Abcam)
- Dulbecco´s Phosphate Buffered Saline (Sigma-Aldrich)
- Hydrogen peroxide 100 volumes >30%, (Fisher Scientific)
- ZytoChemPlusHRP Kit/Rabbit (Zytomed)
- Antibody Diluent (Zytomed)
- Auqatex (Merck)
- Hämalaun sauer nach Mayer (Roth)
- Xylol (Merck)
- Ethanol

**Implementation:**

- positive control/localization: coloncarcinoma, lung carcinoma / cytoplasmic, membrane
- let paraffin drain in the heat cabinet at 72°C for 30min
- 2 x Xylol for 10 min.; 2 x 100%/ 96%/ 70% ethanol for 5 min.; wash in distillated aqua
- samples for 10min. in 3% Hydrogen Peroxide / wash in distillated aqua
- wash samples for 6min. in Dulbecco´s Phosphate Buffered Salin
- samples in protein-block (ZytoChemPlusHRP Kit/Rabbit) for 10min
- wash samples for 6min. in Dulbecco´s Phosphate Buffered Salin
- ad primary antibody UPC1, dilution 1: 200, let it over night at 4°C in AntibodyDiluent
- wash samples for 6min. in Dulbecco´s Phosphate Buffered Salin
- 15min. secondary antibody; ZytoChemPlusHRP Kit/Rabbit
- wash samples for 6min. in Dulbecco´s Phosphate Buffered Salin
- 15min. enzym-konjugat; ZytoChemPlusHRP Kit/Rabbit
- wash samples for 6min. in Dulbecco´s Phosphate Buffered Salin
- AEC dye, microscopic control after 5-15min / stop reaction in distillated aqua
- counter-dyeing and blue with “Mayers Hämalaun”
- cover with “Auqatex“

**CIDEA**

**Reagents:**

- Anti- CIDEA 1 antibody (Abcam)
- Citrate Buffer, (Zytomed)
- Dulbecco´s Phosphate Buffered Saline (Sigma-Aldrich)
- Hydrogen peroxide 100 volumes >30%, (Fisher Scientific)
- ZytoChemPlusHRP Kit/Rabbit (Zytomed)
- Antibody Diluent (Zytomed)
- Auqatex (Merck)
- Hämalaun sauer nach Mayer (Roth)
- Xylol (Merck)
- Ethanol

**Implementation:**

- positive control/localization: skin, fat tissue / cytoplasmic, nucleus
- let paraffin drain in the heat cabinet at 72°C for 30min
- 2 x Xylol for 10 min.; 2 x 100%/ 96%/ 70% ethanol for 5 min.; wash in distillated aqua
- citrate Buffer pH6 for ½ h, cook in a steamer for ½ h
- cool down samples for 20 min.
- samples for 10min. in 3% Hydrogen Peroxide / wash in distillated aqua
- wash samples for 6min. in Dulbecco´s Phosphate Buffered Salin
- samples in protein-block (ZytoChemPlusHRP Kit/Rabbit) for 10min
- wash samples for 6min. in Dulbecco´s Phosphate Buffered Salin
- ad primary antibody CIDEA 1, dilution 1: 1000, let it over night at 4°C in AntibodyDiluent
- wash samples for 6min. in Dulbecco´s Phosphate Buffered Salin
- 15min. secondary antibody; ZytoChemPlusHRP Kit/Rabbit
- wash samples for 6min. in Dulbecco´s Phosphate Buffered Salin
- 15min. enzym-konjugat; ZytoChemPlusHRP Kit/Rabbit
- wash samples for 6min. in Dulbecco´s Phosphate Buffered Salin
- AEC dye, microscopic control after 5-15min / stop reaction in distillated aqua
- counter-dyeing and blue with “Mayers Hämalaun”
- cover with “Auqatex“

**Glut4**

**Reagents:**

- Anti- Glut4 1 antibody (Abcam)
- Citrate Buffer, (Zytomed)
- Dulbecco´s Phosphate Buffered Saline (Sigma-Aldrich)
- Hydrogen peroxide 100 volumes >30%, (Fisher Scientific)
- ZytoChemPlusHRP Kit/Rabbit (Zytomed)
- Antibody Diluent (Zytomed)
- Auqatex (Merck)
- Hämalaun sauer nach Mayer (Roth)
- Xylol (Merck)
- Ethanol

**Implementation:**

- positive control/localization: heart muscle / cytoplasmic
- let paraffin drain in the heat cabinet at 72°C for 30min
- 2 x Xylol for 10 min.; 2 x 100%/ 96%/ 70% ethanol for 5 min.; wash in distillated aqua
- citrate Buffer pH6 for ½ h, cook in a steamer for ½ h
- cool down samples for 20 min.
- samples for 10min. in 3% Hydrogen Peroxide / wash in distillated aqua
- wash samples for 6min. in Dulbecco´s Phosphate Buffered Salin
- samples in protein-block (ZytoChemPlusHRP Kit/Rabbit) for 10min
- wash samples for 6min. in Dulbecco´s Phosphate Buffered Salin
- ad primary antibody Glut4 1 1µg/ml, dilution 1: 900, let it over night at 4°C in AntibodyDiluent
- wash samples for 6min. in Dulbecco´s Phosphate Buffered Salin
- 15min. secondary antibody; ZytoChemPlusHRP Kit/Rabbit
- wash samples for 6min. in Dulbecco´s Phosphate Buffered Salin
- 15min. enzym-konjugat; ZytoChemPlusHRP Kit/Rabbit
- wash samples for 6min. in Dulbecco´s Phosphate Buffered Salin
- AEC dye, microscopic control after 5-15min / stop reaction in distillated aqua
- counter-dyeing and blue with “Mayers Hämalaun”
- cover with “Auqatex“

**MAC387**

**Reagents:**

- Anti- MAC387 antibody (Abcam)
- Citrate Buffer, (Zytomed)
- Dulbecco´s Phosphate Buffered Saline (Sigma-Aldrich)
- Hydrogen peroxide 100 volumes >30%, (Fisher Scientific)
- ZytoChemPlusHRP Kit/Rabbit (Zytomed)
- Antibody Diluent (Zytomed)
- Auqatex (Merck)
- Hämalaun sauer nach Mayer (Roth)
- Xylol (Merck)
- Ethanol

**Implementation:**

- positive control/localization: lymph nodes, colon carcinoma / macrophage
- let paraffin drain in the heat cabinet at 72°C for 30min
- 2 x Xylol for 10 min.; 2 x 100%/ 96%/ 70% ethanol for 5 min.; wash in distillated aqua
- citrate Buffer pH6 for ½ h, cook in a steamer for ½ h
- cool down samples for 20 min.
- samples for 10min. in 3% Hydrogen Peroxide / wash in distillated aqua
- wash samples for 6min. in Dulbecco´s Phosphate Buffered Salin
- samples in protein-block (ZytoChemPlusHRP Kit/Rabbit) for 10min
- wash samples for 6min. in Dulbecco´s Phosphate Buffered Salin
- ad primary antibody CISEA 1, dilution 1: 200, let it over night at 4°C in AntibodyDiluent
- wash samples for 6min. in Dulbecco´s Phosphate Buffered Salin
- 15min. secondary antibody; ZytoChemPlusHRP Kit/Rabbit
- wash samples for 6min. in Dulbecco´s Phosphate Buffered Salin
- 15min. enzym-konjugat; ZytoChemPlusHRP Kit/Rabbit
- wash samples for 6min. in Dulbecco´s Phosphate Buffered Salin
- AEC dye, microscopic control after 5-15min / stop reaction in distillated aqua
- counter-dyeing and blue with “Mayers Hämalaun”
- cover with “Auqatex“

**Myf5**

**Reagents:**

- Anti- Myf5 antibody (Abcam)
- Citrate Buffer, (Zytomed)
- Dulbecco´s Phosphate Buffered Saline (Sigma-Aldrich)
- Hydrogen peroxide 100 volumes >30%, (Fisher Scientific)
- ZytoChemPlusHRP Kit/Rabbit (Zytomed)
- Antibody Diluent (Zytomed)
- Auqatex (Merck)
- Hämalaun sauer nach Mayer (Roth)
- Xylol (Merck)
- Ethanol

**Implementation:**

- positive control/localization: colon carcinoma / intracellular
- let paraffin drain in the heat cabinet at 72°C for 30min
- 2 x Xylol for 10 min.; 2 x 100%/ 96%/ 70% ethanol for 5 min.; wash in distillated aqua
- citrate Buffer pH6 for ½ h, cook in a steamer for ½ h
- cool down samples for 20 min.
- samples for 10min. in 3% Hydrogen Peroxide / wash in distillated aqua
- wash samples for 6min. in Dulbecco´s Phosphate Buffered Salin
- samples in protein-block (ZytoChemPlusHRP Kit/Rabbit) for 10min
- wash samples for 6min. in Dulbecco´s Phosphate Buffered Salin
- ad primary antibody Myf5, dilution 1: 100, let it over night at 4°C in AntibodyDiluent
- wash samples for 6min. in Dulbecco´s Phosphate Buffered Salin
- 15min. secondary antibody; ZytoChemPlusHRP Kit/Rabbit
- wash samples for 6min. in Dulbecco´s Phosphate Buffered Salin
- 15min. enzym-konjugat; ZytoChemPlusHRP Kit/Rabbit
- wash samples for 6min. in Dulbecco´s Phosphate Buffered Salin
- AEC dye, microscopic control after 5-15min / stop reaction in distillated aqua
- counter-dyeing and blue with “Mayers Hämalaun”
- cover with “Auqatex“

**SCA1**

**Reagents:**

- Anti- SCA 1 antibody (Ray Biotech)
- Citrate Buffer, (Zytomed)
- Dulbecco´s Phosphate Buffered Saline (Sigma-Aldrich)
- Hydrogen peroxide 100 volumes >30%, (Fisher Scientific)
- ZytoChemPlusHRP Kit/Rabbit (Zytomed)
- Antibody Diluent (Zytomed)
- Auqatex (Merck)
- Hämalaun sauer nach Mayer (Roth)
- Xylol (Merck)
- Ethanol

**Implementation:**

- positive control/localization: colon carcinoma / intracellular
- let paraffin drain in the heat cabinet at 72°C for 30min
- 2 x Xylol for 10 min.; 2 x 100%/ 96%/ 70% ethanol for 5 min.; wash in distillated aqua
- citrate Buffer pH6 for ½ h, cook in a steamer for ½ h
- cool down samples for 20 min.
- samples for 10min. in 3% Hydrogen Peroxide / wash in distillated aqua
- wash samples for 6min. in Dulbecco´s Phosphate Buffered Salin
- samples in protein-block (ZytoChemPlusHRP Kit/Rabbit) for 10min
- wash samples for 6min. in Dulbecco´s Phosphate Buffered Salin
- ad primary antibody SCA1 dilution 1: 200, let it over night at 4°C in AntibodyDiluent
- wash samples for 6min. in Dulbecco´s Phosphate Buffered Salin
- 15min. secondary antibody; ZytoChemPlusHRP Kit/Rabbit
- wash samples for 6min. in Dulbecco´s Phosphate Buffered Salin
- 15min. enzym-konjugat; ZytoChemPlusHRP Kit/Rabbit
- wash samples for 6min. in Dulbecco´s Phosphate Buffered Salin
- AEC dye, microscopic control after 5-15min / stop reaction in distillated aqua
- counter-dyeing and blue with “Mayers Hämalaun”
- cover with “Auqatex“
